# Whole-Genome Sequence Analysis of an Extensively Drug-Resistant Salmonella enterica Serovar Agona Isolate from an Australian Silver Gull (*Chroicocephalus novaehollandiae*) Reveals the Acquisition of Multidrug Resistance Plasmids

**DOI:** 10.1128/mSphere.00743-20

**Published:** 2020-11-25

**Authors:** Max L. Cummins, Martina Sanderson-Smith, Peter Newton, Nicholas Carlile, David N. Phalen, Kimberly Maute, Leigh G. Monahan, Bethany J. Hoye, Steven P. Djordjevic

**Affiliations:** a The ithree institute, University of Technology Sydney, Ultimo, NSW, Australia; b Australian Centre for Genomic Epidemiological Microbiology, University of Technology Sydney, Broadway, NSW, Australia; c School of Chemistry and Molecular Bioscience and Molecular Horizons, University of Wollongong, Wollongong, NSW, Australia; d Illawarra Health and Medical Research Institute, Wollongong, NSW, Australia; e NSW Health Pathology, Microbiology, Wollongong Hospital, Wollongong, NSW, Australia; f Ecosystems and Threatened Species, NSW Department of Planning, Industry and Environment, Hurstville, NSW, Australia; g Sydney School of Veterinary Sciences, University of Sydney, Sydney, NSW, Australia; h School of Earth, Atmospheric and Life Sciences, University of Wollongong, Wollongong, NSW, Australia; Escola Paulista de Medicina/Universidade Federal de São Paulo

**Keywords:** IncHI2, wildlife, gull, AMR, plasmid, XDR, *Salmonella*, Agona, ESBL, IncX, MDR, One Health, *blaCTX*

## Abstract

Although most of the approximately 94 million annual human cases of gastroenteritis due to Salmonella enterica resolve without medical intervention, antimicrobial therapy is recommended for patients with severe disease. Wild birds can be natural hosts of *Salmonella* that pose a threat to human health; however, multiple-drug-resistant serovars of S. enterica have rarely been described. In 2012, silver gull (Chroicocephalus novaehollandiae) chicks at a major breeding colony were shown to host *Salmonella*, most isolates of which were susceptible to antibiotics. However, multiple-drug-resistant (MDR) Escherichia coli with resistance to carbapenems, ceftazidime, and fluoroquinolones was reported from this breeding colony. In this paper, we describe a novel MDR *Salmonella* strain subsequently isolated from the same breeding colony. SG17-135, an isolate of S. enterica with phenotypic resistance to 12 individual antibiotics but only nine antibiotic classes including penicillins, cephalosporins, monobactams, macrolides, fluoroquinolones, aminoglycosides, dihydrofolate reductase inhibitors (trimethoprim), sulfonamides, and glycylcyclines was recovered from a gull chick in 2017. Whole-genome sequence (WGS) analysis of SG17-135 identified it as Salmonella enterica serovar Agona (*S*. Agona) with a chromosome comprising 4,813,284 bp, an IncHI2 ST2 plasmid (pSG17-135-HI2) of 311,615 bp, and an IncX1 plasmid (pSG17-135-X) of 27,511 bp. pSG17-135-HI2 housed a complex resistance region comprising 16 antimicrobial resistance genes including *bla*_CTX-M-55_. The acquisition of MDR plasmids by S. enterica described here poses a serious threat to human health. Our study highlights the importance of taking a One Health approach to identify environmental reservoirs of drug-resistant pathogens and MDR plasmids.

**IMPORTANCE** Defining environmental reservoirs hosting mobile genetic elements that shuttle critically important antibiotic resistance genes is key to understanding antimicrobial resistance (AMR) from a One Health perspective. Gulls frequent public amenities, parklands, and sewage and other waste disposal sites and carry drug-resistant Escherichia coli. Here, we report on SG17-135, a strain of Salmonella enterica serovar Agona isolated from the cloaca of a silver gull chick nesting on an island in geographic proximity to the greater metropolitan area of Sydney, Australia. SG17-135 is closely related to pathogenic strains of *S*. Agona, displays resistance to nine antimicrobial classes, and carries important virulence gene cargo. Most of the antibiotic resistance genes hosted by SG17-135 are clustered on a large IncHI2 plasmid and are flanked by copies of IS*26*. Wild birds represent an important link in the evolution and transmission of resistance plasmids, and an understanding of their behavior is needed to expose the interplay between clinical and environmental microbial communities.

## Abstract (precis)

Defining environmental reservoirs hosting mobile genetic elements that shuttle critically important antibiotic resistance genes is key to understanding antimicrobial resistance (AMR) from a One Health perspective. Gulls frequent public amenities, parklands, and sewage and other waste disposal sites and carry drug-resistant Escherichia coli.

## INTRODUCTION

*Salmonella* is a zoonotic pathogen comprising over 2,500 serovariants (serovars and serotypes) of which approximately 100 cause human disease (1). Symptoms typical of salmonellosis include diarrhea, fever, and abdominal cramps, and individuals of all ages can be affected (2). Lateral gene transfer plays a significant role in the emergence of epidemic clones that cause repeated episodes of salmonellosis (3, 4). Specifically, the acquisition of plasmids, *Salmonella* pathogenicity islands (SPIs), ColV and related IncF-type virulence plasmids, and antimicrobial resistance (AMR)-imparting single nucleotide polymorphisms (SNPs) represent key mechanisms by which epidemic clones may arise (4). Given the importance of lateral gene transfer events in shaping the evolution of virulent and/or multiple-drug-resistant (MDR) lineages of Salmonella enterica, a genomic microbiological approach that embraces a One Health perspective, recognizing relationships among human, animal, and environmental sources of pathogens (and nonpathogens) (5), is needed to identify reservoirs in which the mobile elements conferring MDR and virulence gene carriage circulate (6).

*Salmonella* Agona is a serovar with the potential to both be multidrug resistant and cause disease. Phylogenetically, *Salmonella* Agona’s clade structure is considered monomorphic (7), with molecular studies suggesting that it emerged in 1932 and underwent global expansion during the 1960s (7). Recombination and the acquisition of phage and other laterally acquired DNA are major drivers of genetic diversity in *S*. Agona (7, 8). This serovar is also known to harbor *Salmonella* genomic island 1 (SGI1) (9), an integrative mobilizable element that is part of a broad family of related elements associated with MDR including, in some cases, resistance to critically important antimicrobials (10). Moreover, strains of *S*. Agona carrying SGI1 are known to cause infections in both humans and poultry (9, 11, 12).

*Salmonella* Agona is increasingly recognized as a cause of foodborne disease outbreaks. In earlier studies, *S*. Agona linked to tea and meat products was responsible for outbreaks in Germany in 2002 to 2003 (77 patients) (13) and in 10 countries (163 infections) across Europe and the United Kingdom in 2008 (14). In 2017, *S.* Agona was one of several S. enterica serovars implicated in four human salmonellosis outbreaks (244 cases) across multiple U.S. states. The causative agent was traced back to contaminated papayas produced in Mexico (15). In 2015, *S.* Agona was also responsible for a small outbreak of gastrointestinal disease that was traced to contaminated sushi in Sydney, Australia (16). In Japan, *S*. Agona has caused outbreaks of disease in humans and has risen to prominence, replacing Salmonella enterica serovar Infantis as the dominant serovar in the poultry industry (17). In Australia, *S*. Agona has (i) been linked to *Salmonella* infections in humans originating from chicken eggs (18), (ii) been isolated from Australian layer hen sheds (19), and (iii) is a significant contaminant of grain in Australian feed mills, where it was the most frequently isolated serovar in raw feed (20), suggesting it may have an environmental reservoir.

The silver gull (Chroicocephalus novaehollandiae) and other gull species globally are important biological indicators of environmental microbial contamination and can carry *Enterobacteriaceae* encoding resistance to antibiotics including third-generation cephalosporins, fluoroquinolones, and carbapenems (21–24). For instance, a 2012 study of gull chicks at three nesting colonies in New South Wales (NSW), Australia, isolated Escherichia coli and other enterobacterial species that carry the metallo-beta-lactamase gene *bla*_IMP-4_ (21). Cloacal carriage of S. enterica was also seen in 13% (66/504) of these chicks, among which 17 serotypes were represented. The majority (56/66) of the *Salmonella* isolates were recovered from chicks at nests on Big Island, Five Islands Nature Reserve, just off the coast of Port Kembla, NSW. However, only a few (3/66) of these S. enterica isolates showed resistance to the panel of antibiotics tested, and those that did displayed resistance to a limited number of antibiotic classes. Notably, genes encoding carbapenem resistance seen in E. coli were not seen in *Salmonella* in the 2012 study (21).

Because of their foraging behavior, gulls have adapted well to coastal urban environments (25). Their gut flora is likely to be influenced by foraging in these environments, including sites where they may acquire drug-resistant *Enterobacteriaceae* such as municipal waste disposal sites and wastewater and sewage plants (26–28). As part of a larger study sampling silver gulls on Big Island that commenced in 2017, we identified several *Salmonella* isolates with reduced susceptibility to a broad range of antimicrobials including those considered critically important to human health. One *S*. Agona isolate, SG17-135, was striking in that it displayed phenotypic resistance to nine antibiotic classes. Here, we undertook whole-genome sequencing (WGS) to characterize the genetic features of strain SG17-135. A hybrid assembly, using a combination of short- and long-read sequencing, provided the first complete genome of an Australian *S.* Agona isolate, which facilitated resolution of the sequences of the chromosome and plasmids residing in SG17-135 and a deeper understanding of the mobile genetic elements that capture and mobilize antimicrobial resistance and virulence-associated genes (VAGs).

## RESULTS AND DISCUSSION

### Phenotypic antibiotic susceptibility testing.

Vitek2 and calibrated dichotomous susceptibility (CDS) assays revealed SG17-135 was resistant to nine different classes of antimicrobials. Specifically, resistance was detected to aminoglycosides, cephalosporins (first to fourth generation), glycylcyclines, macrolides, monobactams, penicillins, quinolones, sulfonamides, and trimethoprim (Table 1). Moreover, the formation of an elliptical pattern of clearing between the amoxicillin-clavulanate disc and both the cefepime and cephalexin discs in the CDS test indicated the production of a plasmid-mediated extended-spectrum beta-lactamase (ESBL) by this isolate (29).

**TABLE 1:** Resistance profiles of *Salmonella* isolate (SG17-135) isolated from a silver gull chick on Five Islands, NSW^*a*^

Antibiotic class	Antibiotic	CDS disc diffusion annular radius (mm)	Vitek2 MIC (μg/ml)
Aminoglycosides	Amikacin	8	≤2 (R)*
	Gentamicin	**0 (R)**	**≥16 (R)**
	Tobramycin	4	8 (R)*

Carbapenems	Ertapenem	10	
	Imipenem	10	
	Meropenem	11	≤0.25

Cephalosporins	Cefepime	**0 (R)**	**≥64 (R)**
	Ceftazidime		**≥64 (R)**
	Cefazolin		**≥64 (R)**
	Cefoxitin		16 (R)*
	Ceftriaxone	**0 (R)**	**≥64 (R)**
	Cephalexin	**0 (R)**	
	Ciprofloxacin	**4 (R)**	2 (I)

Macrolide	Azithromycin	**0 (R)**	

Monobactam	Aztreonam	**0 (R)**	

Nitrofurantoin	Nitrofurantoin		32

Penicillin	Ampicillin	**0 (R)**	**≥32 (R)**

Polymyxin	Polymyxin B	6	

Quinolones	Nalidixic acid	**0 (R)**	
	Norfloxacin		2

Tetracycline	Tigecycline	**2 (R)**	

Dihydrofolate reductase inhibitor	Trimethoprim	**0 (R)**	**≥16 (R)**

Multiple	Amoxicillin-clavulanic acid	8	4
	Piperacillin-tazobactam	7	8
	Ticarcillin-clavulanic acid		32 (I)
	Trimethoprim-sulfamethoxazole	**0 (R)**	**≥320 (R)**

a Profiles were based on CDS agar disc diffusion (annular radius of the zone of inhibition for each antibiotic [millimeters]) and Vitek2 (AST-N246) (MIC). **(R)**, MIC above breakpoints; (I), intermediate susceptibility according to CLSI breakpoints; (R)*, assigned as resistant by Vitek2 Advanced Expert System but with MIC values below the CLSI clinical breakpoints; all other numerical values indicate susceptibility.

### Sequencing and assembly statistics.

Short-read sequencing generated 1,058,312 reads with approximately 31-fold coverage. In regard to long-read sequencing, a 48-h MinION run generated 122,848 reads with a mean read length of 1,727 bp. Three circularized sequences were successfully generated from the hybrid assembly, including the *Salmonella* chromosome comprising 4,813,284 bp, an IncHI2 pMLST2 plasmid (pSG17-135-HI2) of 311,615 bp, and an IncX1 plasmid (pSG17-135-X) of 27,511 bp.

### SG17-135 is closely related to S. enterica serovar Agona strains of human origin.

**FIG 1:**
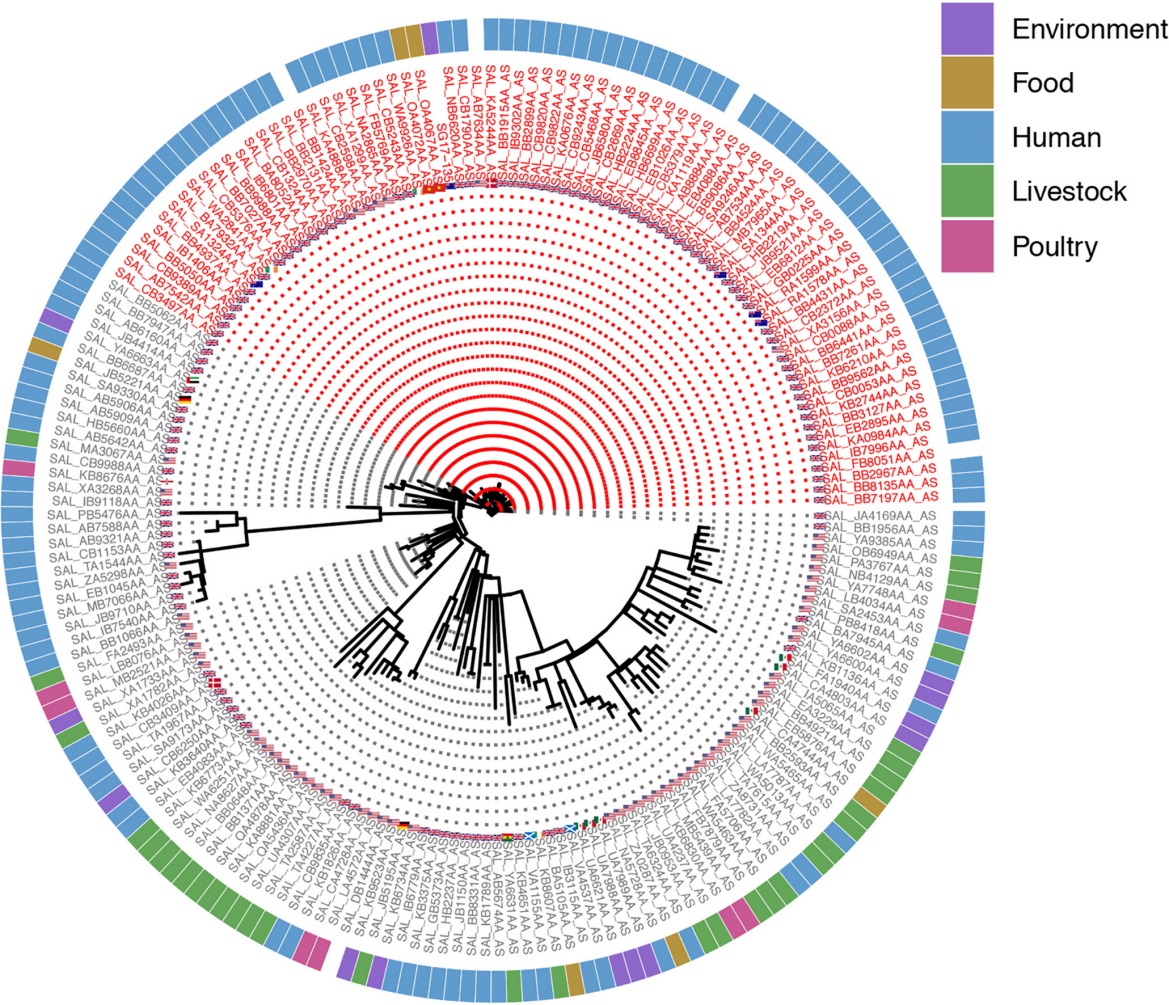
Maximum likelihood tree visualizing phylogenetic relatedness of 195 strains of Salmonella enterica serovar Agona. Strains shown in red-tip labels are of HC5:4181, while gray-tip labels are strains of other HC5 groups. The ring of colored bars surrounding the tree indicates strain sources, while the flags adjacent to tip labels indicate the country of origin. The tree is unrooted, and the reference strain is SG17-135. See Table S1 for more information on the strains in this figure. This figure is best viewed separately in digital format.

Analysis of the core genome of 195 phylogenetically diverse *S.* Agona strains revealed that SG17-135 is part of a clade of predominantly human-associated strains of S. enterica serovar Agona from multiple countries (Fig. 1). Among this broader tree of *S.* Agona isolates, 1,743 variable SNP sites were identified. While certain genomic traits were evident across all (or almost all) *S.* Agona isolates, the clade within which SG17-135 resides appears genotypically and phylogenetically distinct (Fig. 1; see also Table S1 in the supplemental material).

10.1128/mSphere.00743-20.1 TABLE S1 Metadata genotype and accessions. Metadata, genotypic data, and accession numbers for each of the strains under analysis. Download Table S1, TXT file, 0.05 MB. Copyright © 2020 Cummins et al. 2020 Cummins et al. This content is distributed under the terms of the Creative Commons Attribution 4.0 International license.

**FIG 2:**
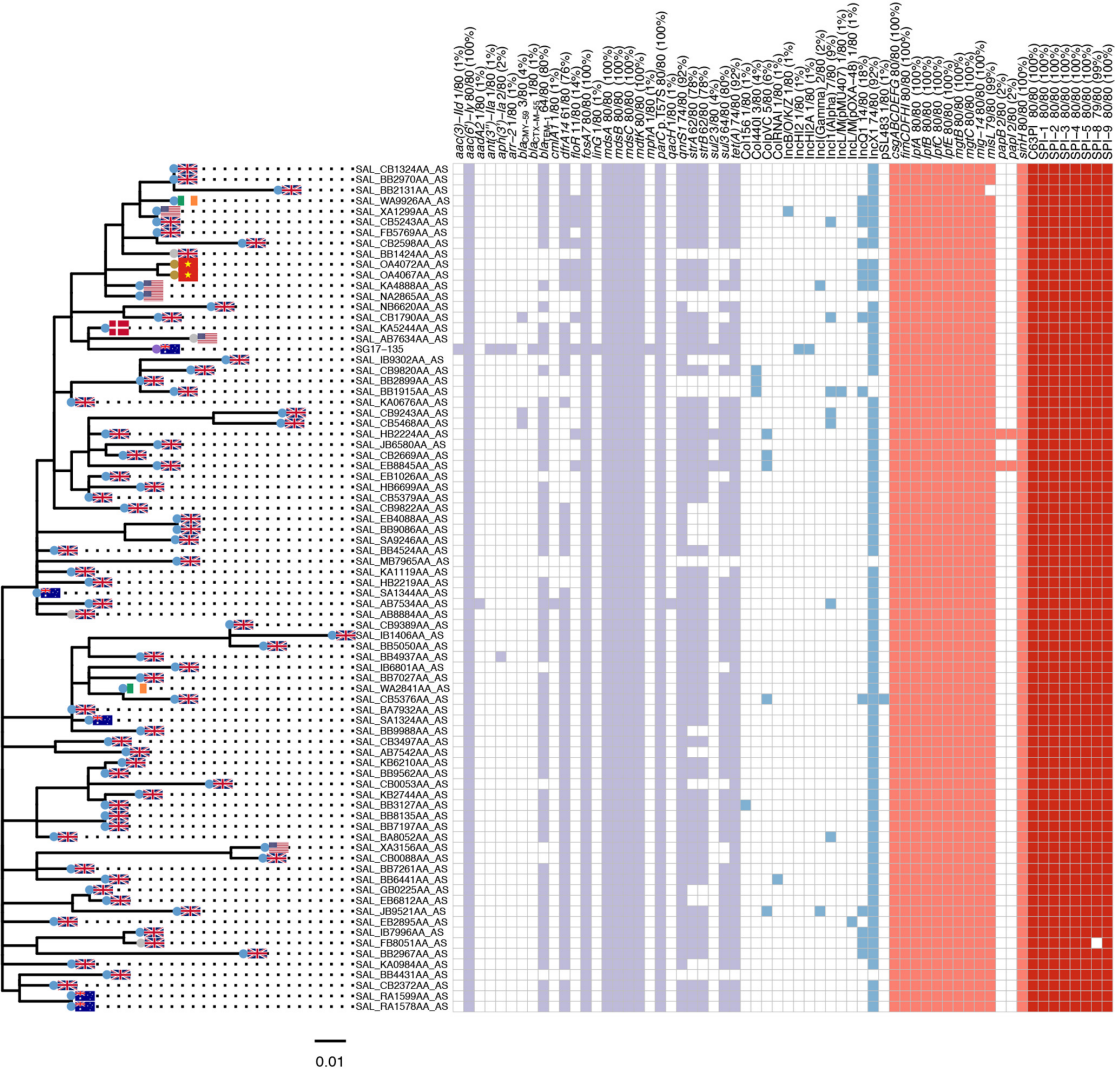
Maximum likelihood tree visualizing phylogenetic relatedness of 80 strains of Salmonella enterica serovar Agona. The genotypic table (right of the tree) indicates the presence or absence of a given gene. Flags on a given tip indicate the country of origin, while colored tips indicate the source of a strain (blue, human; gold, food; purple, environmental; gray, unknown source). The tree is unrooted, and the reference strain is SG17-135. See Table S1 for more information on the strains in this figure.

To explore the traits of this clade in isolation and provide greater phylogenetic resolution, an additional maximum likelihood tree was generated consisting only of the strains more closely related to SG17-135 (Fig. 2). Within this group, isolates exhibit few SNPs across their core genome (Table S2); in total, 289 variable sites were identified across this sublineage, with the most distantly related strain differing from SG17-135 by only 28 SNPs (EnteroBase Assembly barcode: SAL_IB1406AA_AS) (Table S2).

10.1128/mSphere.00743-20.2 TABLE S2 SNP matrix. Pairwise single nucleotide polymorphism distances for strains under analysis. Download Table S2, CSV file, 0.02 MB. Copyright © 2020 Cummins et al. 2020 Cummins et al. This content is distributed under the terms of the Creative Commons Attribution 4.0 International license.

Several human-associated strains are closely related to SG17-135 (EnteroBase barcodes SAL_KA5244AA_AS, SAL_KA0676AA_AS, and SAL_SA1344AA_AS) and differed by less than 10 SNPs. Human strain A64 (SAL_SA1344AA_AS) was isolated from NSW, Australia, in 2016 and differed by only 7 SNPS, a notable observation because SG17-135 was isolated in NSW in 2017 and therefore was of a geographically and temporally similar source as SG17-135. Additionally, save for the *repA*_IncHI2_ and the AMR genes present on pSG17-135-HI2, strains A64 and SG17-135 exhibited identical genotypic profiles. Notably, most strains (73/80; 86%) in Fig. 2 were isolated from humans (Table S1). Clonally related human-associated strains were identified from five different countries; the United Kingdom, Australia, the United States, Ireland, and Denmark. Therefore, SG17-135 is part of a globally disseminated clonal lineage of *S.* Agona predominantly associated with humans.

### SG17-135 carries extensive virulence gene cargo.

As has been previously reported for a broad range of *Salmonella* serovars (30), genes involved in expression of curli fimbriae (*csg*), the product of which is important for host colonization, were identified within SG17-135 and the other Agona strains under analysis more broadly (Table S1). The adhesin-associated long polar fimbria (*lpf*) genes and *Salmonella* pathogenicity islands (SPIs) 1, 2, 3, 4, 5, 8, and 9 were also frequently encountered. (Fig. 1). SPI-1, and to a lesser extent SPI-2, has been demonstrated to play a major role in the pathogenesis of salmonellosis (31, 32). Both of these SPIs, along with SPI-5, contain genes relating to type III secretion systems (T3SSs), while SPI-4 and SPI-9 contain those associated with type I secretion systems (T1SSs), which play important roles in virulence, particularly in delivery of effector proteins into host cells, and host cell adhesion and invasion (33, 34). Taken together, these data indicate the potential for SG17-135 and other Agona strains described here to exhibit virulence in humans, consistent with reports of *S.* Agona as a human pathogen.

### SG17-135—an extensively drug-resistant (XDR) strain among a predominantly MDR lineage.

Among strains closely related to SG17-135, antimicrobial resistance genes *dfrA14* (trimethoprim resistance), *sul3* (sulfonamide resistance), *qnrS1*, *tet(A)*, and *bla*_TEM-1_ (first-generation beta-lactam resistance) were highly represented, with all such genes being carried at rates of 78 to 93%. Notably, AMR genes were far more prevalent in SG17-135 and, to a lesser extent, closely related strains than in the *S*. Agona strains more broadly (Table S1). Notably, with the exception of *bla*_CTX-M-55_ being identified within SG17-135, no other isolates under analysis were found to carry an ESBL gene.

All members of *S*. Agona were found to harbor genes conferring resistance to aminoglycosides and fosfomycin and carry a fluoroquinolone resistance associated SNP (*parC* T57S) and are therefore considered multidrug resistant. However, SG17-135 constitutes the most resistant isolate among those under investigation as it carries 27 distinct genes conferring resistance to nine antibiotic classes including penicillins, cephalosporins, monobactams, macrolides, fluoroquinolones, aminoglycosides, dihydrofolate reductase inhibitors (trimethoprim), sulfonamides, and glycylcyclines.

### Nearly all close relatives of SG17-135 carry plasmids of Inc type X1 but not of IncHI2.

**FIG 3:**
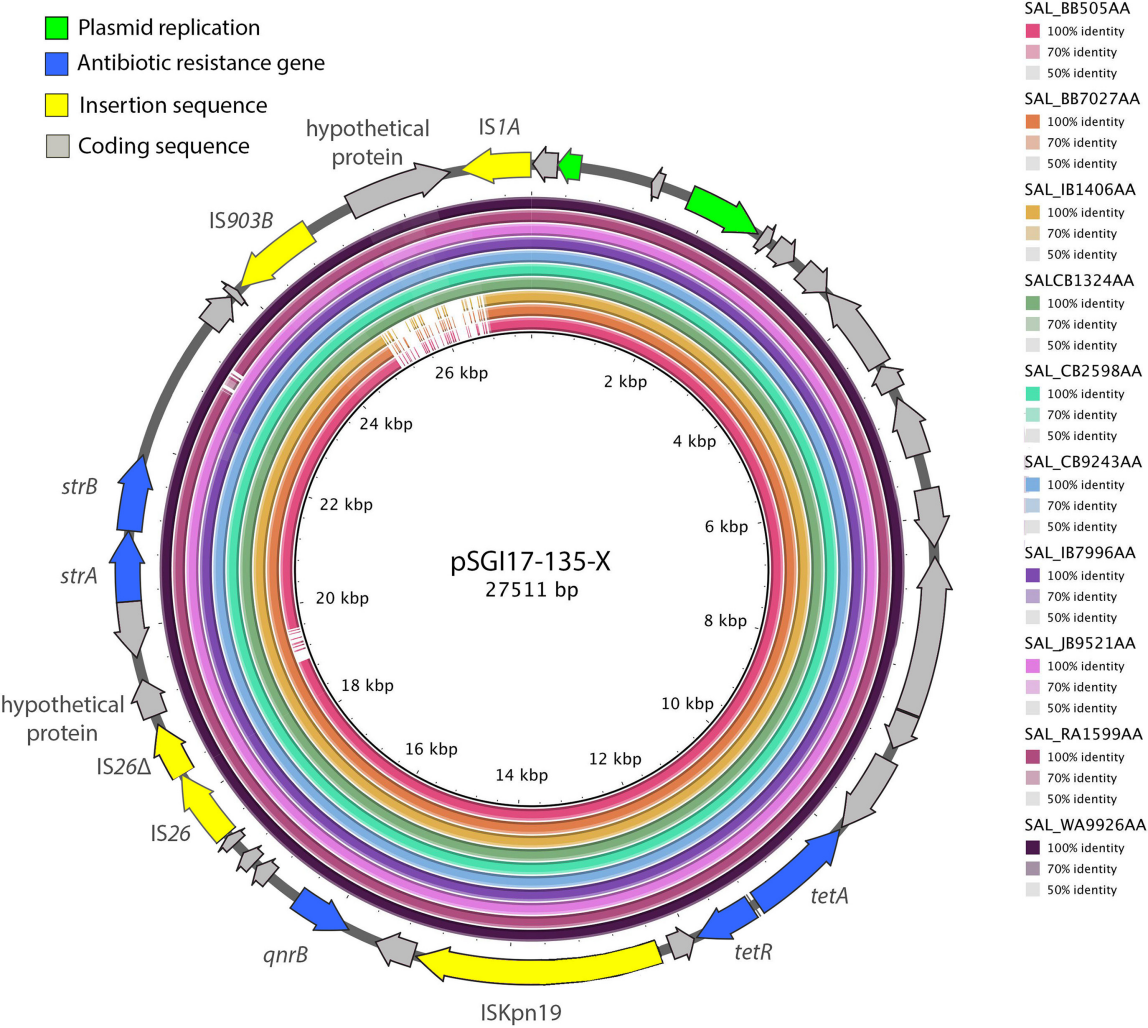
Plasmid alignment generated using BRIG. Ten strains were randomly selected from the same HC5 lineage as SG17-135 and screened for the presence of a plasmid similar to pSGI17-135-X. A schematic of pSG17-135-X can be seen at the outermost ring. Nucleotide identities can be seen in the legend on the right of the image, as can the assembly barcodes for a given sample. See Table S1 for more information on the strains in this figure.

SG17-135 and its close relatives nearly all carry an IncX1 *repA* gene (Fig. 2). Of the IncX1 *repA*-positive members of this clade (72/80 [90%]), almost all (65/72 [90%]) carried an IncX1 *repA* gene, *qnrS1*, and *tet(A)* on the same scaffold (Table S4), providing genetic context for these AMR genes within this group of strains. The most likely interpretation is that an ancestor to the strains depicted in Fig. 2 acquired an IncX1 plasmid carrying *qnrS1* and *tet(A)* prior to clonal expansion. This hypothesis is supported by a BLAST Ring Image Generator (BRIG) alignment of 10 randomly selected *repA*_IncX1_-carrying strains from this clade (Fig. 3), which demonstrates carriage of a pSG17-135-X-like plasmid by all 10 strains. It is also supported by a general absence of IncX1 *repA* carriage by other strains from the broader collection of *S.* Agona strains (Table S1). It should be noted, however, that the IncX1 plasmid gene content varies between strains; while SG17-135 does not carry *sul3* or *bla*_TEM-1_ on pSG17-135-IncX1, other strains exhibit carriage of *sul3* and *bla*_TEM-1_ on the same scaffold as an IncX1 *repA* gene (Table S4). This is to be expected as plasmid resistance gene content is known to be highly dynamic due to the action of mobile genetic elements (35, 36), especially in the presence of IS*26*—an insertion sequence (IS) element which plays important roles in evolution and mobilization of AMR plasmids (35, 37, 38) and one which is present in multiple copies on both pSG17-135-X and pSG17-135-HI2. However, other than SG17-135, no other strains under analysis were found to carry an IncHI2 *repA* gene, highlighting the acquisition of this plasmid as a unique feature of SG17-135.

10.1128/mSphere.00743-20.4 TABLE S4 IncX gene associations. Tabulation of the coscaffolding of *repA* genes (IncX) with AMR genes and other genes of interest. Download Table S4, TXT file, 0.01 MB. Copyright © 2020 Cummins et al. 2020 Cummins et al. This content is distributed under the terms of the Creative Commons Attribution 4.0 International license.

### pSG17-135-HI2 is a highly mosaic multidrug resistance plasmid.

**FIG 4:**
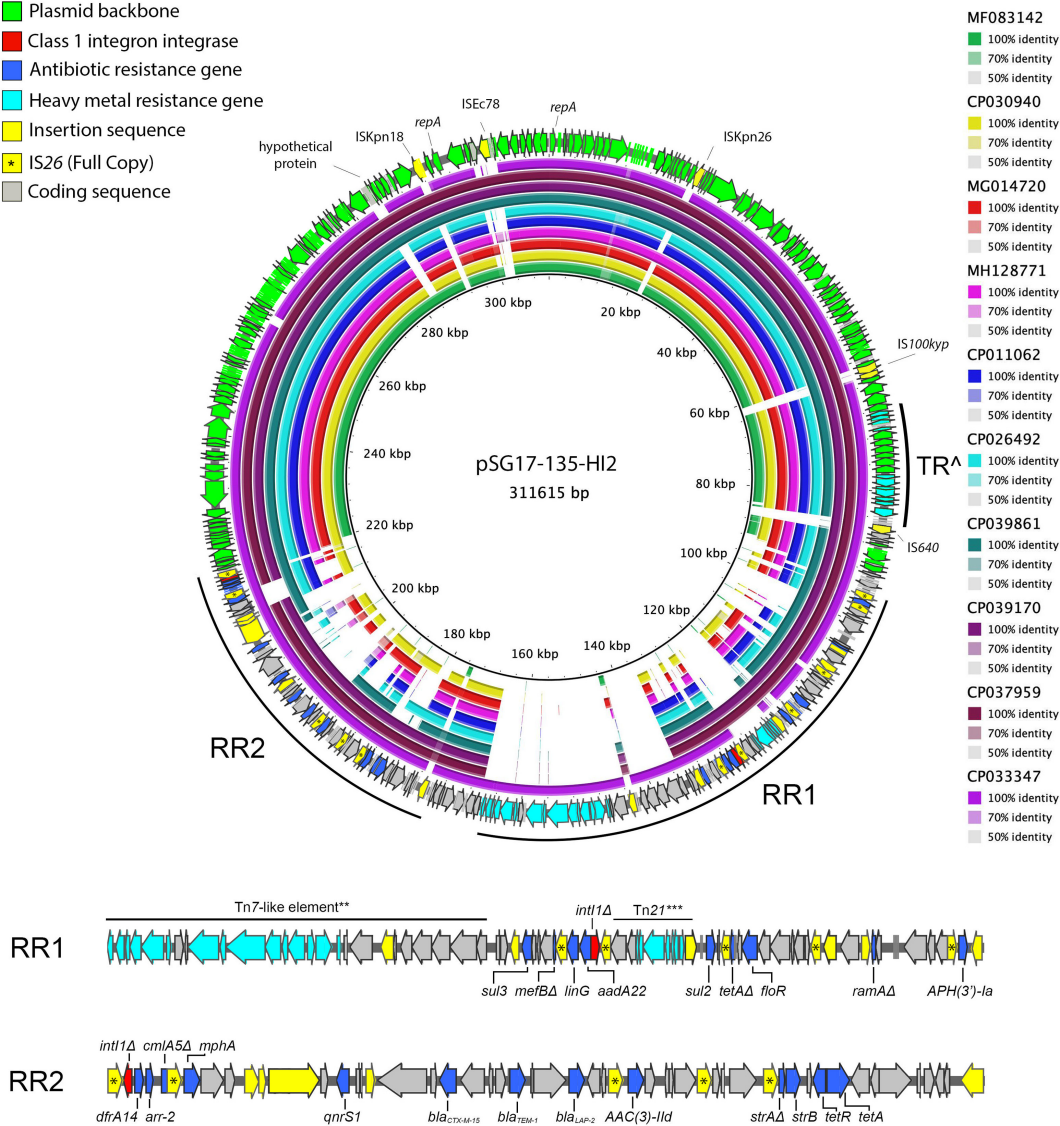
BRIG alignment of the IncHI2 plasmid pSG17-135-HI2 (outer ring) and 10 closely related plasmid sequences (colored internal rings) identified using MEGABLAST analysis of NCBI public databases. Nucleotide identity ranges and strain accession numbers can be seen in the legend on the right of the image. Directionality of arrows indicates the strand location for a given gene, with arrows pointing clockwise being on the top strand and vice versa. RR1/2, resistance region 1/2; **, carries copper resistance operon *cus*; ***, Tn*21*-like element truncated by an IS*6* family element, carries mercury resistance operon *mer*; TR̂, tellurium resistance locus; Δ, truncated gene. More information on the sequences in this figure is available in Table S3.

Of the 26 AMR genes identified in SG17-135 using Abricate, four were localized to pSG17-135-X [*qnrB*, *strA*, *strB*, and *tet(A)*] (Fig. 3), while another six were carried on the chromosome [*mdtK*, *aac(6′)-Iy*, *mdsA*, *mdsB*, *mdsC*, and *fosA7*]. Interestingly, a 1,152-bp hypothetical protein (coordinates 25040 to 26686) was identified on pSG17-135-X which three strains in this BRIG analysis did not carry (Fig. 3). Preliminary analysis using NCBI’s Conserved Domain Database (39) indicates this hypothetical protein is involved in plasmid replication and likely was introduced by the IS*1A* or IS*903B* elements proximal to it. The remaining 16 AMR genes including additional copies of *strA*/*strB* (kanamycin and neomycin resistance) and *tet(A)* (tetracycline resistance), *floR* (florphenicol resistance), *sul2* and *sul3* (resistance to sulfonamides), *ant(3″)-IIa*, *aph(3′)-Ia* and *aac(3)-IId* (aminoglycoside resistance), *linG* (lincosamide resistance), *bla*_TEM-1_ (beta-lactam resistance), *bla*_CTX-M-55_ (ESBL), *qnrS1* (quinolone resistance), *mphA* (macrolide resistance), *arr-2* (rifamycin resistance), and *dfrA14* (trimethoprim resistance) were found to be localized to the HI2 plasmid (Fig. 4), and therefore, its carriage accounted for most of the phenotypic resistance profile of the strain.

10.1128/mSphere.00743-20.3 TABLE S3 IncHI2 plasmid metadata. Metadata on the plasmids used for comparative genomic analysis of pSG17-135-HI2 seen in Fig. 4. Download Table S3, TXT file, 0.01 MB. Copyright © 2020 Cummins et al. 2020 Cummins et al. This content is distributed under the terms of the Creative Commons Attribution 4.0 International license.

pSG17-135-HI2 is an IncHI2-ST2 plasmid that carries a complex and mosaic resistance locus flanked by direct copies of IS*26* that likely has a complex evolutionary history as it is composed of multiple AMR regions that have coalesced through IS- and transposon-mediated integration events, as indicated by the multiple copy number of such elements present throughout this region of the plasmid (Fig. 4). An additional AMR gene on pSG17-135-HI2 was also annotated by RAST as a putative beta-lactamase, with subsequent BLAST analysis determining that the gene shares 100% nucleotide identity with the beta-lactamase *bla*_LAP-2_. *bla*_LAP-2_ is an Ambler class A, narrow-spectrum beta-lactamase that is inhibited by clavulanic acid (40).

Analysis of NCBI databases using MEGABLAST revealed the 10 plasmids most closely related to pSG17-135-HI2 were not of Australian origin. While MDR IncHI2 plasmids have reportedly been isolated within Australian porcine operations (41–44), these plasmids vary in their pMLST types and carry different AMR gene cargo in comparison with pSG17-135-HI2. Instead, this analysis revealed that pSG17-135-HI2 is more closely related to IncHI2 plasmids from China and Taiwan from various sources (Table S3), particularly pCFSA1096 (GenBank accession number CP033347).

pCFSA1096 is an *mcr-1*-carrying plasmid sourced from an isolate of Salmonella enterica serovar London that originated from pork products in China in 2015 (45). Figure 4 shows that pSG17-135-HI2 and pCFSA1096 are highly similar, with BLAST analysis determining they share 99.98% nucleotide identity with a coverage of 92%. Additionally, while the majority of the plasmid backbone is shared between the related plasmids in Fig. 4, only pCFSA1096 and pSG17-135-HI2 carry the copper resistance locus (Fig. 4). Carriage of metal resistance genes is a feature of IncHI2 plasmid sources from *Enterobacteriaceae* derived from the feces of Australian pigs (41–43) and in food animals more broadly (46). Notably, pSG17-135-HI2 appears to have acquired an IS*26*-mobilized fragment of Tn*21*—a structure missing in pCFSA1096 but present in two other IncHI2 plasmids seen in the second and third outermost rings.

Analysis of core genome multilocus sequence typing (cgMLST) schemes in EnteroBase revealed that the chromosomes of strains CFSA1096 and SG17-135 were found to differ by 2,600 to 2,850 core genomic loci. This indicates that while pSG17-135-HI2 and pCFSA1096 share significant sequence identity, the strains hosting these two plasmids are phylogenetically distinct from one another relative to other *Salmonella* strains analyzed, an observation supported by the fact that the host of pCFSA1096 is a Salmonella enterica serovar London strain and the host of pSG17-135-HI2 is a Salmonella enterica serovar Agona strain.

It was also determined by BLAST analysis that the transfer region of pSG17-135-HI2 exhibits 100% coverage and nucleotide identity in comparison with pSal-5364 (GenBank accession number CP039170), an MDR IncHI2 plasmid from a Salmonella enterica serovar Goldcoast outbreak in Taiwan (Fig. 4; see also Table S5). Given that pSal-5364 has been experimentally demonstrated to be conjugative, it is highly probable that pSG17-135-HI2 is also conjugative (47). Other plasmids shown in Fig. 4 with homology to pSG17-135-HI2 were sourced from bacterial hosts of different genera, including Escherichia coli and Citrobacter freundii, highlighting the broad host range of plasmids like pSG17-135-HI2 and the threat that they may pose through circulating between various species also known to be major human pathogens (Table S2).

10.1128/mSphere.00743-20.5 TABLE S5 Plasmid BLAST metrics. Sequence alignment data, including expect values, nucleotide identities, and coverage for alignments visualized in Fig. 3 and 4. Also such metrics for an alignment of the transfer region of pSG17-135-HI2 and a related plasmid, pSal-5364. Download Table S5, TXT file, 1.2 MB. Copyright © 2020 Cummins et al. 2020 Cummins et al. This content is distributed under the terms of the Creative Commons Attribution 4.0 International license.

In conclusion, SG17-135 constitutes a wildlife-derived member of a human-associated, internationally disseminated, virulent, and multidrug-resistant sublineage of S. enterica serovar Agona, an increasingly common serovar associated with human and livestock disease. The acquisition of pSG17-135-HI2 conferred resistance to nine classes of antibiotics in SG17-135 (penicillins, cephalosporins, monobactams, macrolides, fluoroquinolones, aminoglycosides, dihydrofolate reductase inhibitors [trimethoprim], sulfonamides, and glycylcyclines), while pSG17-135-X afforded resistance to streptomycin, quinolones, and tetracycline. Acquisition of this level of resistance is a concern, heightened by the fact that the strain was sourced from a silver gull, a highly mobile (ranging up to 1,000 km) scavenging species observed to frequent sites associated with human waste products (48). Additionally, although this isolate was recovered from a flightless chick (which was found to be *Salmonella* negative 17 days later, before it was capable of flying), silver gulls routinely encounter and interact with humans and urban areas, posing a risk of direct exposure to humans and companion animals. Increasingly, gulls are being recognized as a group of wildlife that can act as a reservoir and transmitter of multiple strains of highly drug-resistant and virulent *Enterobacteriaceae*, as well as the mobile genetic elements associated with such microorganisms (22). This highlights the potential for the transfer of resistance genes on mobile genetic elements between bacterial species in gulls. Future studies should seek to improve knowledge of the foraging behavior of gulls to highlight potential transmission pathways of bacteria and their associated plasmid cargo. Knowledge of the traits and diversity of bacteria found in natural environments and within gulls will also be useful in this regard. This and other studies underscore the need to examine infectious threats to human and animal health through a One Health lens.

## MATERIALS AND METHODS

### Strain isolation and antimicrobial resistance profiling.

Silver gull chicks were sampled at the breeding colony on Big Island, Five Islands Nature Reserve, 900 m off the coast of Port Kembla, NSW (34°29′27″ S, 150°55′42″ E), in October and November of 2017. Cloacal samples were taken using sterile rayon swabs, placed in Amies medium (without charcoal), and stored on ice for up to 24 h prior to culture. Swabs were placed directly into 10 ml selenite F enrichment broth and incubated for 24 h at 37°C before being plated onto MacConkey agar and incubated for a further 18 to 24 h at 37°C. *Salmonella* colonies were differentiated from other Gram-negative bacterial species by harvesting a single, non-lactose-fermenting colony from each plate and inoculating this colony onto a triple sugar iron (TSI) slant and brilliant green agar. Colonies that were glucose positive, produced gas, and were positive for the production of hydrogen sulfide were stored in 10% glycerol LB medium at −80°C. Matrix-assisted laser desorption ionization–time of flight mass spectrometry (MALDI-TOF MS) (Bruker) was used to confirm species identity.

Two different methods were used to screen *Salmonella* isolates for phenotypic antibiotic resistance: the calibrated dichotomous susceptibility (CDS) test (an agar disc diffusion assay) and the Vitek2 (bioMérieux). The CDS test was performed in accordance with the online method manual accessed via the CDS website (http://cdstest.net/) (29). The following antibiotic discs (Oxoid) were tested against a lawn culture of the isolate on SensiTest agar (Oxoid) incubated at 35°C in ambient air: ampicillin (25 μg), ciprofloxacin (2.5 μg), amoxicillin-clavulanate (40/20 μg), ceftriaxone (5 μg), imipenem (10 μg), meropenem (5 μg), cefepime (10 μg), trimethoprim (5 μg), gentamicin (10 μg), piperacillin-tazobactam (50/5 μg), aztreonam (30 μg), trimethoprim-sulfamethoxazole (1.25/23.75 μg), polymyxin B (300 μg), tobramycin (10 μg), amikacin (30 μg), cephalexin (100 μg), ertapenem (10 μg), nalidixic acid (30 μg), azithromycin (15 μg), and tigecycline (15 μg). Plasmid-mediated extended-spectrum beta-lactamase (ESBL) and chromosomal inducible AmpC cephalosporinase were detected as detailed in the CDS method manual (29). The Vitek2 Advanced Expert System (bioMérieux) was used with commercially available Vitek2 AST-N246 cards to determine the MICs of 18 different antibiotics (Table 1) for each *Salmonella* isolate. The CLSI M100-A29 breakpoints were used to determine susceptibility (49).

### Library preparation and whole-genome sequencing.

DNA for short-read sequencing was isolated using the Isolate II genomic DNA kit (Bioline) according to the manufacturer’s instructions. DNA concentration and purity were initially estimated using a NanoDrop 2000c (Thermo Scientific). For library preparation, DNA concentration was determined by Qubit 2.0 fluorometer (Thermo Scientific) and was standardized to 20 ng/μl before using a Nextera DNA library preparation kit. Sequencing was performed using an Illumina NovaSeq generating paired-end reads of 150 bp in length, and demultiplexing was performed using bcl2fastq2 v2.20 on default settings.

A standard protocol incorporating several purification steps using phenol-chloroform/isoamyl alcohol and minimal shearing force was used to isolate genomic DNA used for long-read sequencing as previously described (50). Prior to library preparation, DNA concentration was determined by Qubit 2.0, and its purity was assessed as previously described (50). A genomic sequencing library was prepared from 0.5 μg of genomic DNA using an RBK-004 DNA library preparation kit from Oxford Nanopore Technologies (ONT). DNA purification steps were performed using solid-phase immobilization-reversible beads (Beckman Coulter), and the library was then loaded onto an ONT MinION instrument with a FLO-Min106 (R9.4) flow cell and run for 48 h per manufacturer’s instructions with live base calling disabled.

### Long-read base-calling, demultiplexing, and hybrid assembly.

Reads generated from the long-read sequencing run were base-called using Guppy version 2.3.1 + 1b9405b with command line options “–flowcell FLO-MIN106 –kit SQK-RBK004.” As the sample underwent multiplex sequencing with an additional 11 other isolates, it was necessary to demultiplex the sequence reads prior to genomic assembly. This step was undertaken using qcat version 1.1.0, with the command line options “-k RBK004.” Hybrid genome assembly was undertaken using the Unicycler assembly pipeline version v0.4.6 (51) using default settings.

### Selection of publicly available strains for phylogenetic and genotypic comparisons.

Strains of *S.* Agona used to provide phylogenetic and genotypic context for SG17-135 were identified using cgMLST hierarchical clustering (HC) via EnteroBase v1.1.2 (52, 53). Genomic assemblies of 79 closely related *S.* Agona strains of the same HC5 group as SG17-135 (meaning strains which differ from SG17-135 by five or fewer core genomic loci) were downloaded from EnteroBase for analysis. As EnteroBase houses more than 4,000 *S.* Agona sequences, a subset of 115 additional strains was selected and downloaded for analysis. Code used to select these additional strains can be seen at https://github.com/maxlcummins/SG17-135.

### Genotypic characterization.

Genotyping of SG17-135 and other *S.* Agona genomic assemblies was performed using a custom Snakemake-based workflow which utilized ABRicate (https://github.com/tseemann/Abricate) version 0.9.3 and the nucleotide databases CARD (54), VFDB (55), and PlasmidFinder (56). Additional genotyping of SG17-135 was also performed in this same manner to determine the genetic coordinates of insertion sequences (ISs) and other genomic elements of interest, using the ISfinder database (accessed February 2020) (57) and a separate custom database, respectively. Genes were considered present if a nucleotide identity and coverage threshold of ≥90% was met for a given hit called by ABRicate. The presence of pathogenicity islands was also detected using this Snakemake workflow; however, strains were considered positive for a given pathogenicity island in cases where they exhibited ≥60% coverage and ≥95% nucleotide identity threshold, as is the default setting of SPIfinder (58) (from which SPI nucleotide sequences were sourced). Specific versions of databases and dependencies are available, along with Snakemake workflows, at https://github.com/maxlcummins/SG17-135. Resistance-associated SNPs were detected using PointFinder version 3.1.0 (59).

### Phylogenetic analyses and data visualization.

Phylogenetic analysis was performed using a custom Snakemake-based workflow which performed a full genome alignment of all *S*. Agona strains under analysis (5,152,410 bp in length) using Snippy (60), filtered for recombination using Gubbins (61), extracted variable sites using SNP-sites (62), and subsequently generated a maximum likelihood tree using FastTree2 (63). SG17-135 was used as a reference, and trees were left unrooted. For specific versions of these tools and other dependencies used, as well as the commands used for analysis, please visit https://github.com/maxlcummins/SG17-135. Phylogenetic trees were generated using custom R scripts, which are available at the same GitHub repository, utilizing R packages ggtree 1.16.6 (64), dplyr 0.8.3 (65), ComplexHeatmap 2.0.0 (66), magrittr 1.5 (67), ggplot2 3.2.1 (68), Readr 1.3.1 (69), reshape2 1.4.3 (70), tidytree 0.2.6 (71), and ggimage 0.2.8 (72).

### SG17-135 plasmid annotation and visualization.

Following automatic genome annotation, performed using the RAST tool kit via PATRIC (73) 3.5.43 (https://www.patricbrc.org), custom annotations (informed by abovementioned ABRicate analysis) were imported in SnapGene version 4.1.9 and manually curated. Note that custom annotations were performed only for plasmids pSG17-135-HI2 and pSG17-135-X. Comparative plasmid visualizations were performed using BRIG version 0.95. Sequence alignment metrics produced by BRIG have been made available as supplemental material and at https://github.com/maxlcummins/SG17-135.

### Data availability.

Sequencing reads are available in the Sequence Read Archive (SRA) under BioProject accession number PRJNA606095, wherein long-read sequence data can be accessed under the accession number SRR11069703 and short-read sequence data under the accession number SRR11069704. The complete, assembled genome of SG17-135 is available under NCBI nucleotide accession number CP048775, while custom annotated plasmid sequences for pSG17-135-HI2 and pSG17-135-X can be found under the accession numbers CP048776 and CP048777, respectively. Where publicly available sequences have been utilized, Table S1 in the supplemental material lists their associated sources and accession numbers.
